# *Trichomonas vaginalis* Weakens Human Amniochorion in an In Vitro Model of Premature Membrane Rupture

**DOI:** 10.1155/S1064744995000160

**Published:** 1995

**Authors:** Deborah Draper, Ward Jones, R. Phillip Heine, Michelle Beutz, Janice I. French, James A. McGregor

**Affiliations:** 1 Department of Obstetrics and Gynecology University of Colorado Health Sciences Center Children's Hospital Kempe Research Center Denver CO USA; 2 Magee-Womens Research Institute Department of Obstetrics , Gynecology, and Reproductive Sciences University of Pittsburgh 204 Craft Avenue, Room 530 Pittsburgh PA 15213 USA

## Abstract

*Objective: Trichomonas vaginalis (TV)* infection is associated with preterm rupture of membranes (PROM) and preterm birth. We evaluated the effects of *TV* growth and metabolism on preparations of human amniochorion to understand and characterize how *TV* may impair fetal-membrane integrity and predispose to PROM and preterm birth.

*Methods:* Term fetal membranes were evaluated using an established in vitro fetal-membrane model. Fresh *TV* clinical isolates were obtained from pregnant women. The protozoa (5.0×10^5^ to 1.5×10^6^/ml) were incubated with fetal membranes in modified Diamond's medium for 20 h at 37°C in 5% CO_2_.The effects of fetal-membrane strength (bursting tension, work to rupture, and elasticity) were measured using a calibrated Wheatstone-bridge dynamometer. Tests were also performed to evaluate the effects of 1) inoculum size; 2) metronidazole (50 μg/ml); and 3) cell-free filtrate.

*Results:* The *TV*-induced membrane effects were 1) isolate variable; 2) inoculum dependent; 3) incompletely protected by metronidazole; and 4) mediated by both live organisms as well as protozoan-free culture filtrates. Six of 9 isolates significantly reduced the calculated work to rupture (*P* ≤ 0.02); 7 of 9 reduced bursting tension; and 1 of 9 reduced elasticity. One isolate significantly increased the work to rupture and bursting tension (*P* ≤ 0.002).

*Conclusions:* In vitro incubation of fetal membranes with *TV* can significantly impair the measures of fetal-membrane strength. This model may be used to delineate the mechanisms of *TV*-induced membrane damage. This study suggests that there are enzyme-specific effects as well as pH effects.


Infectious Diseases in Obstetrics and Gynecology 2:267–274 (1995)
© 1995 Wiley-Liss, Inc.

### *Trichomonas vaginalis* Weakens Human Amniochorion in an In Vitro Model of Premature Membrane Rupture

**Deborah Draper, Ward Jones, R. Phillip Heine, Michelle Beutz, Janice I. French, and James A. McGregor**

*Department of Obstetrics and Gynecology, University of Colorado Health Sciences Center, and Children's Hospital, Kempe Research Center, Denver, CO (W.J., M.B., J.I.F., J.A.M.), and Magee-Womens Research Institute, Department of Obstetrics, Gynecology, and Reproductive Sciences, University of Pittsburgh, Pittsburgh, PA (D.D., R.P.H.)*

### Abstract

***Objective:*** ***Trichomonas vaginalis*** **(*TV*) infection is associated with preterm rupture of membranes (PROM) and preterm birth. We evaluated the effects of *TV* growth and metabolism on preparations of human amniochorion to understand and characterize how *TV* may impair fetal-membrane integrity and predispose to PROM and preterm birth.**

***Methods:*** **Term fetal membranes were evaluated using an established in vitro fetal-membrane model. Fresh *TV* clinical isolates were obtained from pregnant women. The protozoa ($5.0 \times 10^5$ to $1.5 \times 10^6$/ml) were incubated with fetal membranes in modified Diamond's medium for 20 h at 37°C in 5% $CO_2$. The effects of fetal-membrane strength (bursting tension, work to rupture, and elasticity) were measured using a calibrated Wheatstone-bridge dynamometer. Tests were also performed to evaluate the effects of 1) inoculum size; 2) metronidazole (50 μg/ml); and 3) cell-free filtrate.**

***Results:*** **The *TV*-induced membrane effects were 1) isolate variable; 2) inoculum dependent; 3) incompletely protected by metronidazole; and 4) mediated by both live organisms as well as protozoan-free culture filtrates. Six of 9 isolates significantly reduced the calculated work to rupture ($P \leq 0.02$); 7 of 9 reduced bursting tension; and 1 of 9 reduced elasticity. One isolate significantly increased the work to rupture and bursting tension ($P \leq 0.002$).**

***Conclusions:*** **In vitro incubation of fetal membranes with *TV* can significantly impair the measures of fetal-membrane strength. This model may be used to delineate the mechanisms of *TV*-induced membrane damage. This study suggests that there are enzyme-specific effects as well as pH effects.**
© 1995 Wiley-Liss, Inc.

Key words

Sexually transmitted disease, fetal membranes, membrane strength, premature rupture of membranes, preterm birth

Preterm birth continues to be the major cause of perinatal morbidity and mortality.[1] Despite advances in neonatal care, there has been little impact on the incidence of preterm birth. This lack of progress in reducing risks of prematurity results from a continuing lack of understanding of the pathobiology of preterm labor and preterm premature rupture of membranes (pPROM).

Much information shows that maternal/reproductive-tract infections as well as host inflammatory responses to infection correlate with a substantial portion of both preterm birth and PROM.[2–6] The mechanisms of these associations have not been elucidated. However, many postulate that virulence factors produced by infecting organisms or by the host in response to infection are

Address correspondence/reprint requests to Dr. Deborah Draper, University of Pittsburgh, Magee-Womens Research Institute, 204 Craft Avenue, Room 530, Pittsburgh, PA 15213.

Received August 23, 1994
Accepted January 25, 1995

the crucial elements.[2] Supporting this theory have been findings that the microorganisms common in vaginal flora, as well as those acknowledged to be genital pathogens, produce enzymes that can weaken fetal membranes in vitro.[7,9] Further, the proteolytic enzymes liberated by both microorganisms and host inflammatory cells can damage fetal membranes, thus decreasing the measurements of bursting tension, work to rupture, and elasticity.[10–12]

*Trichomonas vaginalis (TV)* is a common sexually transmitted protozoan which causes symptomatic exocervicitis and vaginitis as well as asymptomatic infection.[13] *TV* organisms are enzymatically well endowed, producing numerous proteases, hemolytic substances, and other factors which potentially could damage maternal-fetal tissues and predispose infected women to pPROM and preterm birth.[14,15] In a recent large epidemiologic study correlating vaginal infection with pregnancy outcomes, *TV* infection identified at mid-gestation was significantly associated with PROM, preterm birth, and low birth weight.[16,17] Previous studies have demonstrated similar findings.[18] To assess and characterize how trichomoniasis in pregnancy may impair fetal-membrane integrity and increase the risks of PROM, we investigated the ability of *TV* to affect measures of the biomechanical strength of human fetal membranes in a well-characterized in vitro model.

### MATERIALS AND METHODS

### Organism

Nine isolates of *TV*, recovered from pregnant women attending the Denver General Hospital antenatal clinic, were cultured in modified Diamond's medium (Remel Media, Inc., Denver, CO). The organisms were subcultured into fresh Diamond's medium with supplemental antimicrobials (1,000 U of penicillin G/ml, 100 μg/ml of streptomycin, and 5 μg/ml of fungizone) until the *TV* cultures were free of bacterial contaminants (usually 3 passages). The bacterial contamination was assessed by subculture onto chocolate and mycoplasma A7 agar (Remel Media, Lenexa, KS) for 48 h in 5% $CO_2$ at 37°C.

For experiments, axenic protozoa were subsequently subcultured into Diamond's medium without antibiotics for 48 h and tested in late log phase; the protozoan densities ranged from $5 \times 10^5$ to $1.5 \times 10^6$ organisms/ml as determined by a hemocytometer (Neubauer Chamber, Fisher Scientific, Denver, CO). The organisms were left in their used culture media and were not subjected to centrifugation or resuspension. For isolate comparison studies, fresh medium was not added to alter the parasite concentrations due to the negative regulatory effects on virulence-factor expression (Heine and Draper, unpublished observations). Only cultures with >90% parasite motility were applied to the membranes in 0.5-ml aliquots. The dynamometer plates were incubated with 50-rpm shaking (Orbital Shaker Model 361, Fisher Scientific) for 20 h at 37°C in 5% $CO_2$. Uninoculated Diamond's medium was the negative control.

### Membrane Preparation

Our in vitro model of membrane rupture has been described in detail elsewhere.[10,11] For our studies, 17 human fetal membranes were collected aseptically from normal, term placentas delivered by elective cesarean from women who had no evidence of PROM or chorioamnionitis. The membranes were transported to the laboratory for immediate processing. After being washed twice in pseudoamniotic fluid (PAF: 10 mM of urea, 2 mM of glucose, 20 mM of HEPES buffer with 125 mM of NaCl, 7 mM of KCl, 4 mM of calcium lactate, 1.4 mM of $MgSO_4$, and 0.4 mM of $KH_2PO_4$, pH 7.0) to remove blood and debris, the membranes were mounted between 2 sterile Plexiglas plates. These plates have circular perforations which, when aligned and bolted together as a unit, provide exposed surfaces of either amnion or chorion. From 1 membrane, 3 plates were prepared yielding 60 wells/membrane for testing. In this way, the membrane both proximal and distal to the placenta was used. The chorionic side was inoculated with the *TV* culture or control medium, incubated for 20 h, and rinsed. After incubation, yet prior to dynamometer testing, cultures for bacterial contamination were performed on each well. If it was contaminated, the data for that well were eliminated. The amniotic side of each well was tested with a dynamometer to assess the bursting tension, work to rupture, and elasticity. In general, for each test variable, 10–20 replicates were tested on each membrane and 3 membranes were tested for each type of experiment.

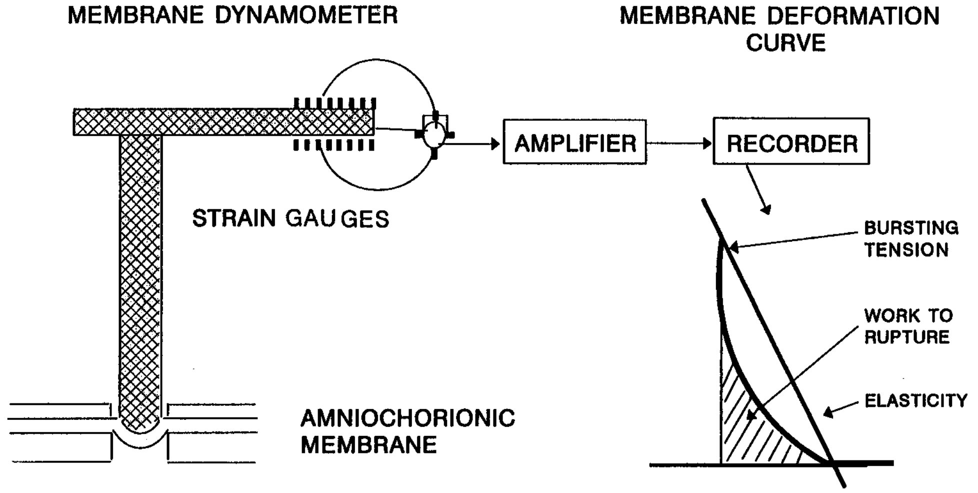

**Fig. 1. Fetal membrane is mounted between 2 Plexiglas plates with holes to form a well in which *TV* is incubated. The membrane dynamometer is constructed from a metal probe mounted on a pressure plate which advances against the membrane until rupture. The pressure plate contains a strain gauge connected to a Wheatstone bridge. The signal is amplified and sent to a strip chart recorder. The membrane deformation curve is analyzed for parameters of bursting strength, elasticity, and work to rupture.**

### Strength Measurement

The dynamometer is constructed from a 3-mm metal probe mounted on a pressure plate which is slowly advanced toward the membrane by a 3-V motor (2.7 mm/min). The pressure plate contains a strain gauge connected to a Wheatstone bridge. The Wheatstone-bridge signal is amplified and sent to the 1-mV strip chart recorder. The probe is advanced against the amniotic side of the membrane until rupture is achieved, and the membrane deformation curve is used to calculate the measures of membrane strength (Fig. 1).

### Metronidazole Protection

For our test of the protective effect of metronidazole, a highly cidal concentration of the drug, which should kill >90% of the isolates, was added to the inocula of parasites and incubated on the membranes. Four strains were chosen for study on 2 different membranes. The inocula were prepared from young, log-phase (40 h old) *TV* cultures at $5 \times 10^5$ organisms/ml; cidal concentrations of 50 μg/ml of metronidazole were added. The age of the culture and the density of parasites were chosen at $5 \times 10^5$ to ensure parasite growth, thus sensitivity to the antibiotic. In Diamond's medium, the maximum growth achievable was 1–2 $\times 10^6$ organisms/ml. Drug-free parasite inocula were prepared as positive controls, and parasite-free, drug-supplemented Diamond's medium was used as a negative control.

### pH Effects

As parasites grow in Diamond's medium (initial pH 6.8), there is usually a concomitant decrease in the pH of the culture medium. The pH may drop as much as 2 pH units in the course of reaching maximum culture density. For our test of the effect of this pH shift on membrane strength as distinguished from the effects of parasite virulence factors, aliquots of uninoculated Diamond's medium were adjusted with concentrated acetic acid, filter-sterilized through a 0.45-μm filter and added to the membranes. The pH of uninoculated Diamond's medium was varied and applied. The inocula varied from pH 4.0 to 7.0 in 0.5-pH increments. The strength of the membranes was measured after incubation for 20 h. The pH effect was tested on 3 different membranes.

### Supernatant Studies

Additional experiments aimed at separating and distinguishing parasite effects from those of secreted virulence factors were performed using culture filtrates. After parasite growth, the pH of the culture was adjusted with 0.1 M of NaOH to pH 6.5 to eliminate pH effect, then split into 2 parts. One portion was filtered and inoculated onto fetal membranes. The effect of this inoculum was compared with the parasite-containing culture. To prepare parasite-free filtrates, 10 ml of a 48-h culture in log phase was centrifuged at 750*g* for 10 min. The supernatant was removed and filtered through a 0.45-μm filter (E-D Scientific Specialists, Intermountain Scientific, Salt Lake City, UT).

### Protease Assay

The protease activity in culture supernatants was assessed by a fluorescent substrate cleavage assay. Fifty microliters of 0.4% resorufin-labeled casein (Sigma Chemical Co., St. Louis, MO) was mixed with a 100-μl aliquot of culture supernatant and 50 μl of incubation buffer (0.2 M Tris with 1 mM of $CaCl_2$, pH 7.8) and incubated for 3 h at 37°C. The reaction was stopped with 500 μl of 5% trichloroacetic acid. The mixture was incubated for 10 min at 37°C and centrifuged at 2,600 rpm in a Beckman table-top centrifuge for 10 min. Four hundred microliters of supernatant was added to 600 μl of assay buffer (0.5 M Tris, pH 8.8) and the mixture read on a Perkin Elmer (Norwalk, CT) M2600 fluorescence spectrophotometer at an excitation wavelength of 578 nm and emission wavelength of 592 nm. Purified trypsin (Sigma Chemical Co.) was used as a standard to construct the activity curves.

### Data Analysis

A Gateway 2000 PC computer and Sigma Scan program (Jandel Scientific, Corte Madera, CA) were used for the calculation of work to rupture (from area under the curve), bursting tension (from peak height), and elasticity/plasticity (from slope of the peak).[10,11] Statistical tests were performed on averaged data from 10 to 20 replicates for each test inoculum or medium controls/membrane. Additionally, the location of the test replicates was randomized across all test plates to normalize intramembrane variability. Experiments were performed on 2–3 different membranes for each isolate. The data from wells containing control medium were compared with data from *TV*-inoculated wells. The data are presented as relative percents of effect rather than grams of work. This normalization is necessary so that the effects can be compared between membrane experiments. The statistics were

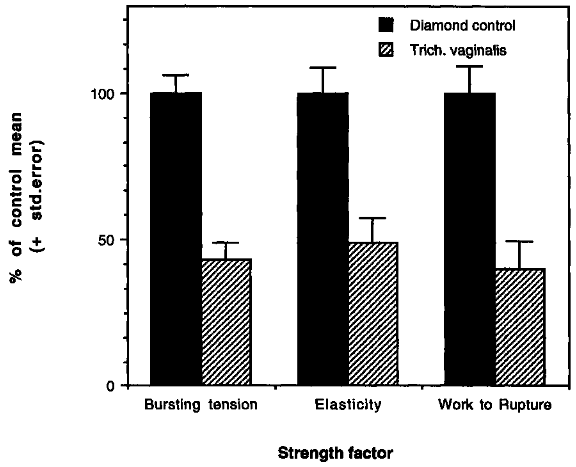

**Fig. 2.** *TV* at $1 \times 10^6$ organisms/ml incubated on human fetal membranes for 20 h. The membranes were tested for tensile strength and work required to rupture the membrane. Overall, *TV* weakened the membrane significantly and reduced the work to rupture by 60% ($P < 0.004$), bursting strength by 57% ($P < 0.002$), and elasticity by 51% ($P < 0.004$).

performed using the Statview PC program (Calabasas, CA). Where data were quantitative and normally distributed, the Student's t-test was used. The Kolmogorov-Smirnov test was used to analyze the significance of nonparametric data with 0.05 as alpha.

### RESULTS

Experiments with a fresh clinical isolate of *TV* demonstrated that this protozoan was capable of significantly weakening human fetal membranes in this model. Work to rupture was decreased by 60% ($P < 0.004$) (Fig. 2). Decreasing concentrations of organisms correlated with decreasing membrane damage (data not shown). When several isolates from pregnant women were tested, the parasite effects occurred in an isolate-and dose-dependent manner (Table 1). In general, most strains that achieved a concentration of $1 \times 10^6$ organisms/ml had the ability to significantly impair membrane strength. However, at a concentration of $5 \times 10^5$ organisms/ml, there was evidence of isolate variation in the ability to reduce measures of fetal-membrane strength. For example, strains $T_5$, $T_8$, and $T_9$ had identical inoculum densities, yet 1 had a significant effect on strength while the other 2 did not (Table 1). The data in Table 1 are normalized and presented as percent effect rather than as change

**TABLE 1. Percent decrease in measures of fetal-membrane strength in the presence of *TV***

| | | % | | |
|---|---|---|---|---|
| *TV* strain | Inoculum (organisms/ml) | Bursting tension (*P*) | Work to rupture (*P*) | Elasticity (*P*) |
| $T_1$ | $1.5 \times 10^6$ | 57 (0.0002) | 60 (0.004) | 51 (0.002) |
| $T_2$ | $5.0 \times 10^5$ | 54 (0.002)[a] | 64 (0.004)[a] | 1 (NS)[b] |
| $T_3$ | $7.7 \times 10^5$ | 28 (0.01) | 37 (0.01) | 3 (NS) |
| $T_4$ | $5.0 \times 10^5$ | 18 (0.01) | 19 (0.067) | 6 (NS) |
| $T_5$ | $9.0 \times 10^5$ | 18 (0.10) | 25 (0.10) | 10 (NS) |
| $T_6$ | $1.0 \times 10^6$ | 47 (0.001) | 59 (0.0003) | 5 (NS) |
| $T_7$ | $1.3 \times 10^6$ | 43 (0.001) | 57 (0.002) | 2 (NS) |
| $T_8$ | $9.0 \times 10^5$ | 34 (0.01) | 37 (0.02) | 9 (NS) |
| $T_9$ | $9.0 \times 10^5$ | 23 (0.05) | 25 (0.14) | 1 (NS) |

[a]Note that stain $T_2$ increased the bursting tension and work to rupture of this membrane.
[b]NS = not significant.

in units of force because there can be substantial variation between membranes. For example, for negative-control inoculated membranes, the values of work to rupture ranged from 4,797 ± 1,458 to 11,402 ± 4,551 $mm^2$/time, bursting tensions from 168 ± 26 to 265 ± 38 g, and elasticity's slopes for negative controls from 4.12 ± 0.65 to 6.31 ± 1.05. For membranes inoculated with *TV* cultures, the work effects ranged from 2,804 ± 1,567 to 9,261 ± 2,724 $mm^2$/time, bursting tensions from 96 ± 28 to 259 ± 60 g, and elasticities from 2.56 ± 0.55 to 5.89 ± 0.93. Across a membrane surface (or within a single membrane), the variability could range from as little as 10% to as much as 40%.

The effects of metronidazole treatment on *TV*-induced membrane weakening were observed. Metronidazole killed trichomonads as evidenced by a 10- to 100-fold drop in hemocytometer counts ($1.0 \times 10^6$ to $1.5 \times 10^4$) for all strains after 20 h incubation. Metronidazole treatment only partially protected the membranes from parasite attack. The effect of strain $T_6$ was significantly reduced ($P < 0.05$). There was a tendency toward protection with the 3 remaining isolates ($P = 0.07$). This finding suggested that live parasites were required for maximal membrane damage to occur and that virulence factors were preformed and secreted in the culture supernatant (Fig. 3).

Garber and Bowie[19] have suggested that the *TV*-induced acidic pH shift may directly damage cultured cells. Experiments were performed to control for the expected decrease in the culture medium's pH accompanying trichomonal growth. To sum-

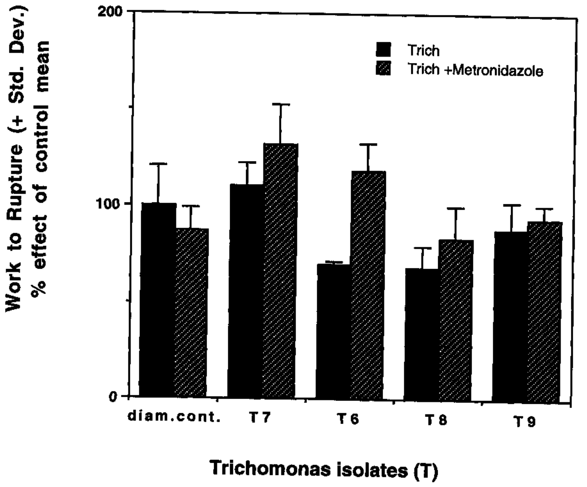

**Fig. 3.** Metronidazole's protective effect on fetal-membrane strength in the presence of *TV*. Cultures of 4 isolates of *TV* were incubated on fetal membranes with and without metronidazole (50 μg/ml) for 20 h. Metronidazole treatment tended to increase work to rupture. Tests with isolate $T_6$ demonstrated significant protection ($P < 0.05$), whereas the others showed a trend toward protection ($P = 0.07$). diam. cont. = Diamond's medium control.

marize, no significant weakening was seen in 2 of 3 membranes at pH values from 5.0 to 7.0. The other membrane showed a 30% reduction in all parameters at pHs ≤6.0. All 3 membranes showed significant weakening at pH ≤4.5 ($P < 0.05$). No membranes showed weakening at pH 6.5 or 7.0. While significant, the weakening attributable to pH effect was less than that attributable to *TV* (data not shown). This suggests that pH does affect the membrane strength, but does not account for all of the observed impairment associated with *TV* growth.

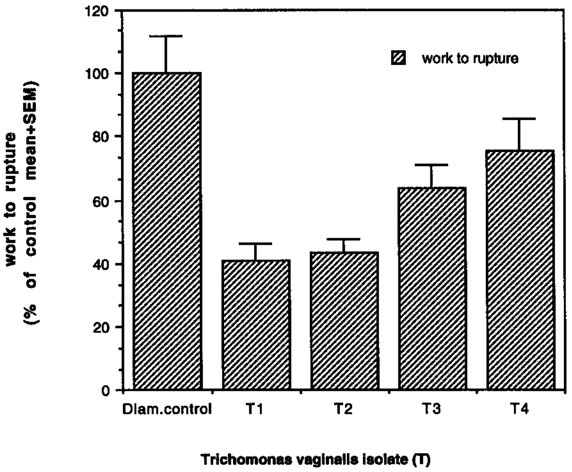

Fig. 4. Culture supernatants of *TV* impair measures of membrane strength. Culture supernatants of *TV* grown in Diamond's (Diam.) medium for 5 days were filtered through a 0.45-μm filter with pH adjusted to 6.5, applied to fetal membranes, and tested in the usual manner. All supernatants significantly weakened fetal membranes and decreased bursting tension ($P < 0.010$).

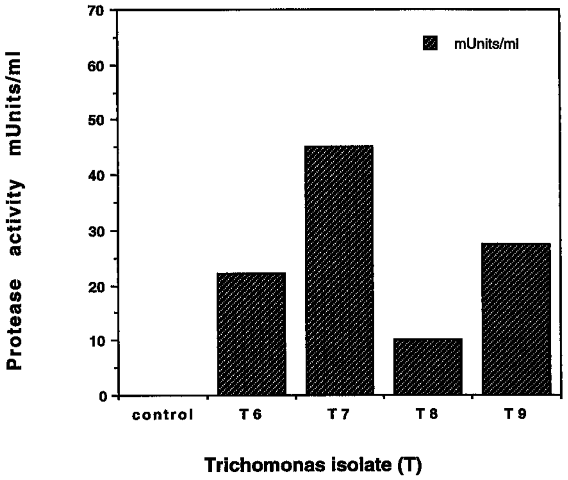

Fig. 5. Protease activity in cell-culture supernatants of *TV*. Four protozoan isolates from pregnant women were grown in modified Diamond's medium and centrifuged, and the supernatant was filtered through a 0.45 μm filter. The filtrates were assayed in triplicate and expressed as a mean of protease activity relative to a trypsin standard.

Tests also were performed to assess the role of extracellular products of *TV* metabolism on membrane damage. Cell-free filtrates from 48-h log-phase parasites significantly decreased the fetal-membrane bursting tension and work to rupture, suggesting that extracellular factors produced by *TV* can impair fetal-membrane strength (Fig. 4). Preliminary studies aimed at identifying the virulence factor demonstrated the presence of proteases in the culture supernatants. Protease activities ranged from 10 mU activity/ml of supernatant to 44 mU/ml. The level of protease activity did not entirely correlate with the amount of membrane damage (Fig. 5).

### DISCUSSION

*TV* can significantly impair human fetal-membrane strength in an established in vitro model by reducing the measures of bursting tension, work to rupture, and rarely elasticity. At lower test inocula, *TV* isolates from pregnant women damaged the fetal membranes in a strain-variable manner. These effects can be attributed to several factors including 1) numbers of viable parasites; 2) secretion of membrane-damaging molecules; and 3) pH effects.

The inoculum comparison study (Table 1) suggests that parasite burden is important in pathogenesis. Philips et al.[20] showed that 70% of women with trichomoniasis have $\geq 10^4$ organisms/ml in their vaginal secretions. There are no data for parasite burdens in pregnant women. The estimates of vaginal densities in nonpregnant women range from $10^4$ to $10^5$ and occasionally to $10^6$ organisms/ml, which approximate the test inocula in this study.[20] For our studies, we used clinically relevant test inocula from actively growing organisms. Inocula were not adjusted to equivalent densities for each isolate due to the negative regulatory effects of fresh media on virulence-factor expression (Heine and Draper, unpublished observations). It is important to realize that the parasites were applied to membranes in their used (spent) culture media, which contained live organisms, as well as secreted factors, and that both of these factors appear to contribute to trichomonal virulence.

In Table 1, the appearance of one isolate that increased bursting tension and work to rupture is difficult to explain. It may be that this strain produces a large amount of denaturation of membrane protein and actually causes the membrane to toughen. The basis of this is not clear. Our membrane studies of strength and pH sensitivity suggest that membranes weaken as pH drops. However, we did not test pH values below 4.0. In laboratory studies of parasite growth, the pH decrease usually ranges from 6.5 to 5.0, although we have seen as low as 4.0. The organism does not survive for long below pH 5.0 and rapidly dies and lyses. However, the lysing organism liberates hydrogenosomes which are rich in acidic metabolic products.[21] This observation may explain the phenomenon of membrane toughening for this isolate.

In the metronidazole studies, protection was not significant in 3/4 tests, although a trend was seen toward protection ($P = 0.07$). The failure to provide full protection may in part be explained by inoculum age, density, and the presence of preformed virulence enzymes that are not sensitive to the antibiotic. We chose a test inoculum of $5 \times 10^5$ organisms/ml and an inoculum age of 40 h. This strategy was necessary for several reasons. This culture density was chosen so that the inoculum allowed additional growth to the maximum density achievable in Diamond's medium ($1–2 \times 10^6$ organisms/ml). The culture age provided parasites that were still in log-phase growth and presumably sensitive to the antibiotic. However, a culture age of 40 h means that there has been time to release some secreted factors. This timing was necessary because protozoa placed into fresh medium do not damage membranes within 20 h and do not produce virulence enzymes in Diamond's medium for at least 24 h until presumably all of their nutritional needs are met (Draper, unpublished observations). Finally, strain variability in the production of secreted virulence factors may explain failures of significant metronidazole protection.

Our data indicate that parasite-free culture filtrates are capable of damaging membranes even when corrected for pH. The fact that *TV* produces extracellular cytotoxic factors suggests that *TV* need not be directly present in order to damage amniochorion or other tissues. Honigberg et al.[22] and others[23,24] have demonstrated ultrastructural changes at a distance from trophs in tissue biopsies of vaginal epithelium during trichomoniasis, suggesting the presence of diffusible, extracellular virulence factors. Conversely, Alderate and Pearlman[25] and other workers[24,26] using tissue-culture systems have suggested that parasite contact is required for cytotoxicity. Garber et al.[27] have identified a cell detaching factor (CDF), which is a secreted, high-molecular-weight protease, and have shown that purified CDF is capable of disturbing cell monolayers in the absence of parasites. Additionally, our metronidazole studies show that a portion of the effect is due to viable growing parasites. The observations of Garber et al.[27] and Honigberg et al.[22] coupled with our findings of membrane damage from trichomonal supernatants suggest that diffusible, membrane-damaging factors are produced and could damage fetal membranes overlying the cervical os.

Our preliminary analysis of the factors in culture supernatant indicates that proteases are present. Presumably, these virulence factors attack the components of membranes that engender strength and resist rupture. These components are probably collagen fibers and fiber bundles. It has been suggested that the dense matrix of collagen fibers underlying the basement membrane of the amnion is the main "load-bearing" structure.[28] Proteases which are collagenases or gelatinases (attack denatured collagen) would be obvious enzymes of virulence, and it is known that this protozoan is protease rich.[14,15] It is also known that trichomonal proteases are expressed in vivo and that antibodies are produced in response to protease expression during infection.[29]

Finally, the observation of parasite variability in impacting fetal-membrane strength has clinical relevance. It suggests that not all *TV* strains are alike and may explain why all pregnant women with undetected trichomoniasis do not suffer from PROM. In vivo, the membrane damage required for PROM may be an interaction of the number of protozoa in the vaginal canal, liberated virulence factors in the upper genital tract, virulence of the specific parasite, and host response factors.

In this in vitro model, clinical isolates of *TV* impaired the measures of fetal-membrane strength in a strain-variable and inoculum-dependent manner. Membrane damage was partially prevented by metronidazole treatment. Further basic research and clinical investigation are required to better evaluate the mechanisms of *TV*-associated effects that can increase the risks of pPROM and preterm birth.

### REFERENCES

1. McCormick MC: The contribution of low birthweight to infant mortality and childhood morbidity. N Engl J Med 321:82–87, 1985.
2. McGregor JA: Prevention of preterm birth: New initiatives based on microbial host interactions. Obstet Gynecol Surv 43:1–14, 1988.
3. Gibbs RS, Romero R, Hillier SL, Eschenbach DA, Sweet

RL: A review of premature birth and subclinical infection. Am J Obstet Gynecol 166:1515–1528, 1992.

4. Naeye RL, Peters EC: Causes and consequences of premature rupture of fetal membranes. Lancet 1:192, 1980.
5. Minkoff H, Grunebaum AN, Schwarz RH, et al.: Risk factors for prematurity and premature rupture of membranes: A prospective study of vaginal flora in pregnancy. Am J Obstet Gynecol 150:965, 1984.
6. Knox IC, Hoerner JK: The role of infection in premature rupture of membranes. Am J Obstet Gynecol 59:190, 1950.
7. McGregor JA, French JI, Reller LB, Todd JK, Makowski EL: Adjunctive erythromycin treatment for idiopathic preterm labor: Results of a randomized, double-blinded, placebo-controlled trial. Am J Obstet Gynecol 154(1):98–103, 1985.
8. McGregor JA, Lawellin D, Franco-Buff A, Todd JK, Makowski EL: Protease production by microorganisms associated with reproductive tract infection. Am J Obstet Gynecol 154(1):109–114, 1986.
9. McGregor JA, Lawellin D, Franco-Buff A, Vasil M, Todd JK: Phospholipase C production by microorganisms associated with female upper genital tract infection. Society for Gynecologic Investigation, Phoenix, AZ, March 20–23, 1985.
10. McGregor JA, Schoonmaker JN, Lawellin DW, Lunt B: Prevention of microbial induced impairment of amniochorion by specific protease inhibitor. Society for Perinatal Obstetricians, New Orleans, LA, March 3–6, 1989.
11. Schoonmaker JN, Lawellin DW, Lunt BD, McGregor JA: Bacteria and inflammatory cells reduce chorioamnionic membrane integrity and tensile strength. Obstet Gynecol 74:590–596, 1989.
12. McGregor JA, French JL, Lawellin D, Franco-Buff A, Smith C, Todd JK: In vitro study of bacterial protease induced reduction of chorioamnionic membrane strength and elasticity. Obstet Gynecol 69:167–174, 1986.
13. Rein MF: Clinical manifestation of urogenital trichomoniasis in women. In Honigberg BM (ed): Trichomonads Parasitic in Humans. New York: Springer-Verlag, pp 225–234, 1989.
14. Neale KA, Alderete JF: Analysis of the proteinases of representative *Trichomonas vaginalis* isolates. Infect Immun 58:157–162, 1990.
15. Lockwood BC, North MJ, Scott KI, Bremner AF, Coombs GH: The use of a highly sensitive electrophoretic method to compare the proteinases of trichomonads. Mol Biochem Parasitol 24:89–95, 1987.
16. Cotch MF, Pastorek JG, Nugent RP, Yeng DE, Martin DH, Eschenbach DA: Demographic and behavioral predictions of *Trichomonas vaginalis* infection among pregnant women. Obstet Gynecol 78:1087–1092, 1991.
17. Cotch MF, Pastorek JG: Effect of *Trichomonas vaginalis (TV)* carriage on pregnancy outcome. Presented at the International Society for Sexually Transmitted Diseases, 9th International Meeting, Banff, Alberta, Canada, October 6–9, 1991.
18. Hardy PH, Hardy JB, Nell EE, et al.: Prevalance of six sexually transmitted disease agents among pregnant inner city adolescents and pregnancy outcomes. Lancet 2:333–337, 1984.
19. Garber GE, Bowie WR: The effect of *Trichomonas vaginalis* and the role of pH on cell culture monolayer viability. Clin Invest Med 13(2):71–76, 1990.
20. Philips A, Carter-Scott P, Rodgers C: An agar culture technique to quantitate *Trichomonas vaginalis* from women. J Infect Dis 155:304–308, 1987.
21. Muller M: Biochemistry of *Trichomons vaginalis*. In Honigberg BM (ed): Trichomonads Parasitic in Humans. New York: Springer-Verlag, pp 53–83, 1989.
22. Honigberg BM, Gupta PK, Spence MR, Frost JK, Kuczynska K, Choromanski L, Warton A: Pathogenicity of *Trichomonas vaginalis:* Cytopathologic and histopathologic changes of the cervical epithelium. Obstet Gynecol 64: 179–184, 1984.
23. Gupta PK, Frost JK: Cytopathology and histopathology of the female genital tract in *Trichomonas vaginalis* infection. In Honigberg BM (ed): Trichomonads Parasitic in Humans. New York: Springer-Verlag, pp 274–290, 1989.
24. Lushbaugh WB, Turner AC, Gentry GA, Klykken PC: Characterization of a secreted cytoactive factor from *Trichomonas vaginalis*. Am J Trop Med Hyg 41:18–28, 1989.
25. Alderete JF, Pearlman E: Pathogenic *Trichomonas vaginalis* cytotoxicity to cell culture monolayers. Br J Vener Dis 60:99–105, 1984.
26. Krieger JN, Ravdin JI, Rein MF: Contact-dependent cytopathogenic mechanisms of *Trichomonas vaginalis*. Infect Immun 50:778–786, 1985.
27. Garber GE, Favel-Lemchuk LT, Bowie WR: Isolation of a cell detaching factor of *Trichomonas vaginalis*. J Clin Microbiol 27:1548–1553, 1989.
28. Skinner S, Campos G, Higgins G: Collagen content of human amniotic membranes: Effect of gestational length and premature rupture. Obstet Gynecol 57:487–492, 1981.
29. Alderete JF, Newton E, Dennis C, Neale KA: Antibody in sera of patients infected with *Trichomonas vaginalis* is to trichomonad proteinases. Genitour Med 67:331–334, 1991.

## References

[PDF_00693] Alderete J. F., Newton E., Dennis C., Neale K. A. (1991). Antibody in sera of patients infected with Trichomonas vaginalis is to trichomonad proteinases.. Genitourin Med.

[PDF_00680] Alderete J. F., Pearlman E. (1984). Pathogenic Trichomonas vaginalis cytotoxicity to cell culture monolayers.. Br J Vener Dis.

[PDF_00644] Cotch M. F., Pastorek J. G., Nugent R. P., Yerg D. E., Martin D. H., Eschenbach D. A. (1991). Demographic and behavioral predictors of Trichomonas vaginalis infection among pregnant women. The Vaginal Infections and Prematurity Study Group.. Obstet Gynecol.

[PDF_00657] Garber G. E., Bowie W. R. (1990). The effect of Trichomonas vaginalis and the role of pH on cell culture monolayer viability.. Clin Invest Med.

[PDF_00686] Garber G. E., Lemchuk-Favel L. T., Bowie W. R. (1989). Isolation of a cell-detaching factor of Trichomonas vaginalis.. J Clin Microbiol.

[PDF_00593] Gibbs R. S., Romero R., Hillier S. L., Eschenbach D. A., Sweet R. L. (1992). A review of premature birth and subclinical infection.. Am J Obstet Gynecol.

[PDF_00653] Hardy P. H., Hardy J. B., Nell E. E., Graham D. A., Spence M. R., Rosenbaum R. C. (1984). Prevalence of six sexually transmitted disease agents among pregnant inner-city adolescents and pregnancy outcome.. Lancet.

[PDF_00666] Honigberg B. M., Gupta P. K., Spence M. R., Frost J. K., Kuczyńska K., Choromański L., Wartoń A. (1984). Pathogenicity of Trichomonas vaginalis: cytopathologic and histopathologic changes of the cervical epithelium.. Obstet Gynecol.

[PDF_00604] KNOX I. C., HOERNER J. K. (1950). The role of infection in premature rupture of the membranes.. Am J Obstet Gynecol.

[PDF_00683] Krieger J. N., Ravdin J. I., Rein M. F. (1985). Contact-dependent cytopathogenic mechanisms of Trichomonas vaginalis.. Infect Immun.

[PDF_00640] Lockwood B. C., North M. J., Scott K. I., Bremner A. F., Coombs G. H. (1987). The use of a highly sensitive electrophoretic method to compare the proteinases of trichomonads.. Mol Biochem Parasitol.

[PDF_00676] Lushbaugh W. B., Turner A. C., Gentry G. A., Klykken P. C. (1989). Characterization of a secreted cytoactive factor from Trichomonas vaginalis.. Am J Trop Med Hyg.

[PDF_00587] McCormick M. C. (1985). The contribution of low birth weight to infant mortality and childhood morbidity.. N Engl J Med.

[PDF_00629] McGregor J. A., French J. I., Lawellin D., Franco-Buff A., Smith C., Todd J. K. (1987). Bacterial protease-induced reduction of chorioamniotic membrane strength and elasticity.. Obstet Gynecol.

[PDF_00607] McGregor J. A., French J. I., Reller L. B., Todd J. K., Makowski E. L. (1986). Adjunctive erythromycin treatment for idiopathic preterm labor: results of a randomized, double-blinded, placebo-controlled trial.. Am J Obstet Gynecol.

[PDF_00612] McGregor J. A., Lawellin D., Franco-Buff A., Todd J. K., Makowski E. L. (1986). Protease production by microorganisms associated with reproductive tract infection.. Am J Obstet Gynecol.

[PDF_00590] McGregor J. A. (1988). Prevention of preterm birth: new initiatives based on microbial-host interactions.. Obstet Gynecol Surv.

[PDF_00600] Minkoff H., Grunebaum A. N., Schwarz R. H., Feldman J., Cummings M., Crombleholme W., Clark L., Pringle G., McCormack W. M. (1984). Risk factors for prematurity and premature rupture of membranes: a prospective study of the vaginal flora in pregnancy.. Am J Obstet Gynecol.

[PDF_00598] Naeye R. L., Peters E. C. (1980). Causes and consequences of premature rupture of fetal membranes.. Lancet.

[PDF_00637] Neale K. A., Alderete J. F. (1990). Analysis of the proteinases of representative Trichomonas vaginalis isolates.. Infect Immun.

[PDF_00660] Philip A., Carter-Scott P., Rogers C. (1987). An agar culture technique to quantitate Trichomonas vaginalis from women.. J Infect Dis.

[PDF_00625] Schoonmaker J. N., Lawellin D. W., Lunt B., McGregor J. A. (1989). Bacteria and inflammatory cells reduce chorioamniotic membrane integrity and tensile strength.. Obstet Gynecol.

[PDF_00689] Skinner S. J., Campos G. A., Liggins G. C. (1981). Collagen content of human amniotic membranes: effect of gestation length and premature rupture.. Obstet Gynecol.

